# Investigating the Mechanisms of Combined Therapy for MCF-7 Breast Cancer Cells Using Arsenic Trioxide and Resveratrol through Network Pharmacology

**DOI:** 10.1007/s43657-025-00218-9

**Published:** 2025-12-19

**Authors:** Hongye Hu, Yanzhi Wu, Jingwei Hong, Huiying Wang, Xiaohua Zhang, Guanli Huang, Xiaofang Zhu

**Affiliations:** 1 Department of Breast Surgery, The First Affiliated Hospital of Wenzhou Medical University, Wenzhou, 325000 China; 2 Department of Rheumatology, The First Affiliated Hospital of Wenzhou Medical University, Wenzhou, 325000 China; 3 Department of Thyroid Surgery, The First Affiliated Hospital of Wenzhou Medical University, Wenzhou, 325000 China; 4 Department of Rheumatology, Affiliated Dongyang Hospital of Wenzhou Medical University, Dongyang, 322100 China

**Keywords:** Arsenic trioxide, Resveratrol, Apoptosis, Breast cancer, Network pharmacology

## Abstract

Breast cancer (BC) remains the predominant form of cancer among women. Arsenic trioxide (ATO), an element in traditional Chinese medicine, has shown potential for treating BC, particularly when combined with resveratrol. However, the exact mechanisms of their combined action are not fully understood. This study aims to clarify their combined mechanisms through network pharmacology and experimental validation. In vitro experiments confirmed that compared with either agent alone, the combination of ATO and resveratrol more effectively inhibited the production of *estrogen receptor 1* (*ESR1*), *tumor protein P53* (*TP53*), and *v-AKT murine thymoma viral oncogene homolog 1* (*AKT1*) in MCF-7 cells. Our findings indicate that the combination of ATO and resveratrol significantly promotes cell apoptosis by suppressing *ESR1*, *TP53*, and *AKT1*. Thus, ATO and resveratrol together may offer a promising strategy for BC treatment.

## Introduction

Breast cancer (BC) remains the most prevalent carcinoma among women worldwide, with approximately 2.1 million new diagnoses in 2018 alone (Bray et al. 2018). Despite advancements in surgery, radiotherapy, and chemotherapy, BC claimed the lives of 626,679 women in the same year, highlighting the urgent need for new therapeutic strategies (Bray et al. 2018).

Arsenic trioxide (ATO), a conventional remedy in Chinese medicine, has historically been used for over 2000 years due to its anti-inflammatory and antimicrobial properties (Chen et al. 2023a; Paul et al. 2023; Yan et al. 2024). Recently, ATO has gained recognition for its significant anti-cancer properties (Chen et al. 2023b; Mao et al. 2023), particularly in the treatment of acute promyelocytic leukemia (APL) (Hofmann et al. 2020; Zhang et al. 2001), However, while ATO has shown considerable efficacy against BC, it is generally less responsive compared to APL (Chow et al. 2004; Wang et al. 2011; Xin et al. 2020).

Resveratrol, a naturally occurring phytoalexin found in grapes, white hellebore, peanuts, and red wine, has been extensively studied for its anti-aging and anti-inflammatory properties (Lalani et al. 2023; Lu and Serrero 1999; Schmidt et al. 2020; Signorelli and Ghidoni 2005; Varoni et al. 2016). Research has indicated that resveratrol may enhance the therapeutic effects of ATO against various cancer models, including BC (Gu et al. 2015; Mondal et al. 2018; Zhao et al. 2014). The specific mechanisms of action remain unclear.

Network pharmacology is an emerging field that utilizes systems biology and network analysis to systematically evaluate drug effects and interactions (Hopkins 2007, 2008). By mapping the complex biological networks involved in drug action, network pharmacology allows for a more comprehensive understanding of therapeutic mechanisms, thereby improving drug screening and target identification in clinical trials (Hopkins 2008; Jiao et al. 2023).

This study aims to investigate the combined effects of ATO and re﻿sveratrol on BC using network pharmacology techniques. By exploring the potential mechanisms underlying their synergistic action, we seek to uncover innovative therapeutic strategies that can address the shortcomings of existing treatments and improve outcomes for BC patients.

## Materials and Methods

### Anticipating Possible Targets of ATO and Resveratrol in the Course of Treating BC

The necessary target information was gathered from the "biological assay results" section, and UniProt was utilized to gather UniProt IDs. Furthermore, target information regarding ATO and resveratrol was obtained from TargetNet (Yao et al. 2016). It is worth mentioning that targets with a predicted probability of zero were omitted from the analysis. GeneCards (Stelzer et al. 2016) (https//www.genecards.org/, version 5.3) was used to gather BC-related data by searching for 'breast cancer'. Inclusion was limited to targets that had a relevance score of 20 or higher. The targets of ATO and resveratrol, as well as the targets related to BC, were intersected to identify potential targets for treating BC. The resulting targets were then imported into VENNY2.1 for visualization.

### Building the Protein–Protein Interaction (PPI) Network

Through the mapping of ATO, resveratrol, and BC targets, we identified common targets for ATO, resveratrol, and BC. Subsequently, a PPI network was established using STRING (Szklarczyk et al. 2021). The resulting biological network was visualized with the help of Cytoscape software (Shannon et al. 2003).

### Functional Enrichment of Shared Targets for ATO, Resveratrol, and BC

We employed gProfiler31 (https//biit.cs.ut.ee/gprofiler/gost) for the purpose of functional analysis. After making corrections for an FDR of less than 5%, we analyzed enrichments for Gene Ontology (GO) and Kyoto Encyclopedia of Genes and Genomes (KEGG).

### Cells

Our MCF-7 cells (ER^+^ , PR^+^) were obtained from the National Collection of Authenticated Cell Cultures in Shanghai, China, and were used between passages 10 and 20. The cells were grown in RPMI 1640 medium with 10% serum from fetal calves (MilliporeSigma, Burlington, MA, USA), along with penicillin (100 U/mL) and streptomycin (100 μg/mL). The presence of Mycoplasma in the cells was ruled out using DAPI staining with 4′,6-diamidino-2- phenylindole. The cell culture procedure was carried out at a steady 37 °C, within an environment composed of 5% carbon dioxide.

### Approaches for Evaluating Cell Vitality and Proliferation

We first placed MCF-7 cells at a density of 2 × 10^3^ cells per well on 96-well plates and let them attach overnight. Then, we treated the cells with ATO and resveratrol, sourced from MilliporeSigma and Aladdin respectively. ATO was dissolved in a 2% sodium hydroxide solution and resveratrol was prepared by dissolving it in DMSO. The pH was adjusted to seven using hydrochloric acid. We used varying concentrations for the treatment. After 48 h, we used a Cell Counting Kit-8 (C0039, Beyotime, China) to assess the number of viable cells, as per the manufacturer's instructions. Each experimental condition was repeated six times.

In our study, we used GraphPad's Prism 8.4 software to determine the IC50 values for ATO and resveratrol. The IC50 value indicates the concentration required for 50% inhibition. The necessary concentrations for ATO and resveratrol are shown in Fig. 2a and b.

### Terminal Deoxynucleotidyl Transferase Mediated dUTP Nick-End Labeling (TUNEL) Assay

TUNEL analysis was performed to evaluate the apoptosis of MCF-7 cells. The test was conducted following the guidelines provided by the manufacturer (C10618, Invitrogen, Carlsbad, CA, USA). Microscope was used to capture images with green fluorescence that were positive for TUNEL. We counted the positive cells and determined the number and proportion, and then presented the data as the average proportion of positive cells.

### Real-Time Reverse Transcription-Quantitative Polymerase Chain Reaction (RT-qPCR)

TRIzol^®^ Reagent (15596026CN, Invitrogen, Carlsbad, CA, USA) was used to extract total RNA from the cultured cells of each group. The First Strand cDNA Synthesis kit (R211-01, Vazyme Biotech, Nanjing, China) was used to synthesize complementary (c)DNA from 2 μg of total RNA. Subsequently, the cDNA was examined using the SYBR™ Green Realtime PCR kit (Q131-02, Vazyme Biotech). The LightCycler^®^ 96 system (Roche, Basel, Switzerland) was utilized for RT-qPCR, with each reaction performed in triplicate in 96-well plates. PCR specificity was verified by conducting analysis of melting curves following amplification. The manufacturer instructions were followed when using all kits. The primer pairs detailed in Table 1 were used to amplify the cDNAs of *estrogen receptor 1* (*ESR1*), *tumor protein P53* (*TP53*), *v-AKT murine thymoma viral oncogene homolog 1* (*AKT1*)*, Epidermal Growth Factor Receptor* (*EGFR*)*, tumor necrosis factor-alpha* (*TNF-α*)*, Heat shock protein 90 kDa alpha* (*HSP90AA*), *hypoxia-inducible factor 1A* (*HIF1A*), and *β-actin.*

**Table 1:** Sequences for primers

Primer	Sequence
ESR1	Sense 5'- AACCGAGATGATGTAGCCAGC −3'
	Antisense 5'- CAGGAACCAGGGAAAATGTG −3'
TP53	Sense 5'- GCTTGCCACAGGTCTCCC −3'
	Antisense 5'- GAGGCAAGCAGAGGCTGG −3'
AKT1	Sense 5'- AGCGACGTGGCTATTGTGAAG −3'
	Antisense 5'-GCCATCATTCTTGAGGAGGAAGT −3'
EGFR	Sense 5′-CCAAGGCACGAGTAACAAGC-3′;
	Antisense 5'- TCCCAAGGACCACCTCACAG −3'
TNF-α	Sense 5′-AATAGGTTTTGAGGGCCATG-3′; Antisense 5'-TCATCTGGAGGAAGCGGTAG-3'
HSP90AA	Sense 5′-CAGGAGATGGTTAAACACTAG-3′; Antisense 5'-TGGACACTAAGAGAACACAT-3'
HIF1A	Sense 5′-CGTTCCTTCGATCAGTTGTC-3’ Antisense 5'-TCAGTGGTGGCAGTGGTAGT-3’
β-actin	Sense 5'- CATGTACGTTGCTATCCAGGC −3'
	Antisense 5'- CTCCTTAATGTCACGCACGAT −3'

### Western Blotting

MCF-7 cells underwent treatment with ATO (3 μM) and resveratrol (53 μM) for a duration of 48 h. As a control, phosphate-buffered saline was used under the same conditions. Following this, cell lysates were gathered, and a western blotting procedure was performed as per the instructions provided by the manufacturer.

We started by adding the protein samples in 10 μL volumes to each well. We set the voltage at 60 V for 35 min and later bumped it up to 120 V for the next 60 min. It's crucial to keep the voltage stable throughout the electrophoresis process. We made sure the transfer was done at a low temperature. Afterwards, we placed the polyvinylidene difluoride (PVDF) membranes (IPVH00010, Merck Millipore, Shanghai, China) on the gel surfaces. We then blocked the PVDF membranes with 5% milk (D8340, Solarbio, Beijing, China) powder for 2 h at room temperature with a shaker. After a 10-min wash of the membranes with TBST (Servicebio, Wuhan, China), we let them incubate with the primary antibody reaction solution overnight at 4 °C.

We used antibodies, all diluted to 1:2000 and sourced from Abcam, Cambridge, UK. These included the rabbit monoclonal (E91) targeting Estrogen Receptor alpha (ESR) (ab32396), rabbit monoclonal (EP2109Y) targeting AKT1 (ab81283), Mouse monoclonal [G59-12] targeting TP53 (ab308609), and mouse monoclonal (6C5) targeting GAPDH (used as a loading control; ab8245).

After washing the membranes with TBST three times for 10 min each, they were incubated for 2 h with a secondary antibody reaction solution. After another three rounds of 10-min washes with TBST, the results were captured by a Tennant (Tanon 3500, Shanghai, China) series fully automated gel image analysis system. We used Image Lab 3.0 software (Bio-RAD, Inc., Hercules, CA, USA) to quantify the protein bands.

### Statistical Analyses

We processed the data using SPSS22 software. The mean ± standard error of the mean (SEM) displays the results. To compare multiple groups, we employed a one-way analysis of variance (ANOVA). To compare between groups, we applied the Tukey post-hoc test (with a 5% significance level)). We considered results statistically significant when *p* < 0.05.

## Results

### Identification of Potential Action Targets of ATO and Resveratrol in BC

**Fig. 1:**
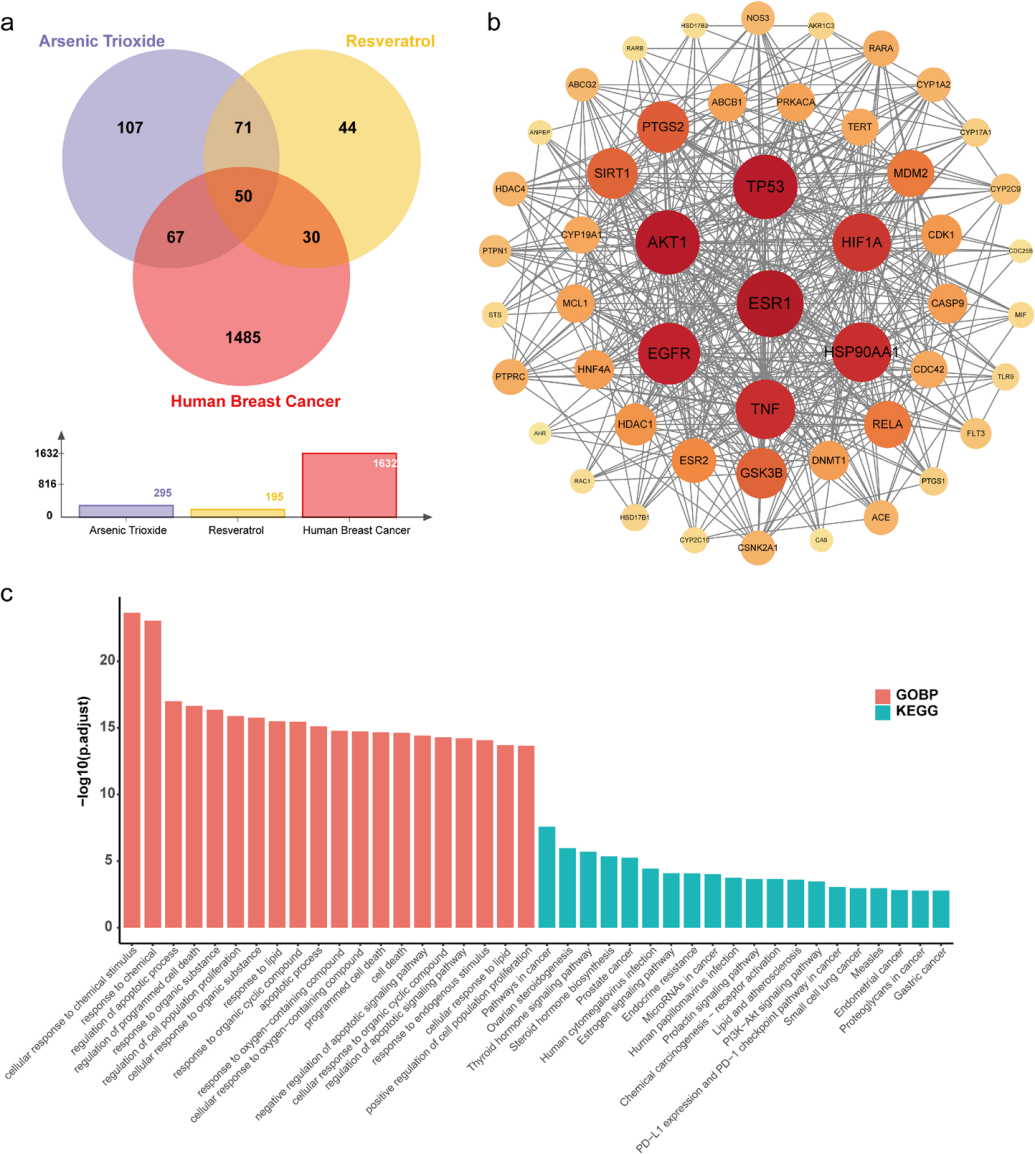
Venn diagram, PPI network, and pathway enrichment analysis of potential ATO and resveratrol targets in BC treatment. **a** A Venn diagram showcasing potential targets of ATO and resveratrol in BC treatment; **b** a network of shared targets in the PPI system, where nodes represent proteins and edges indicate protein–protein associations; and **c** an analysis of pathway enrichment for common targets. The bar graph in section **c** displays the 20 most enriched GO and KEGG pathways from the analysis of 50 shared targets, with the horizontal axis showing the pathways and the vertical axis indicating the enrichment score (− log10 adjusted *p*-value). Each bar represents a specific pathway, with its height reflecting the degree of enrichment

We acquired a total of 1632 targets linked to BC from the GeneCards database. Additionally, we retrieved target information for ATO and resveratrol from TargetNet, with 295 and 195 targets respectively. Figure 1a shows the core genes for further investigation, which were obtained as a total of 50 overlapping targets (Table 2, Table S1).

**Table 2:** Analysis of network topology for targets (top 20 degrees)

No.	Targets	Degree	Average shortest path length	Closeness centrality	Neighborhood connectivity	Radiality
1	ESR1	41	1.163	0.860	19.098	0.996
2	TP53	39	1.204	0.831	19.872	0.995
3	AKT1	39	1.204	0.831	19.410	0.995
4	EGFR	37	1.245	0.803	19.811	0.994
5	HSP90AA1	35	1.286	0.778	20.857	0.993
6	TNF	35	1.286	0.778	20.514	0.993
7	HIF1A	34	1.306	0.766	21.324	0.993
8	PTGS2	29	1.408	0.710	21.483	0.990
9	SIRT1	28	1.429	0.700	23.357	0.990
10	GSK3B	28	1.429	0.700	23.357	0.990
11	MDM2	25	1.490	0.671	24.760	0.988
12	RELA	25	1.490	0.671	23.880	0.988
13	ESR2	23	1.531	0.653	23.304	0.987
14	HDAC1	21	1.571	0.636	26.333	0.986
15	CDK1	20	1.612	0.620	25.550	0.985
16	HNF4A	19	1.612	0.620	25.789	0.985
17	CASP9	19	1.612	0.620	27.474	0.985
18	DNMT1	19	1.633	0.612	26.526	0.985
19	ABCB1	18	1.633	0.612	26.389	0.985
20	MCL1	18	1.633	0.612	27.444	0.985

### Construction of the PPI Network

To fully understand the intricate relationships among the 50 common targets identified, we judiciously assembled a PPI network using STRING. We only considered interactions with a combined score of ≥ 0.9 to guarantee dependability. This network provides a visual depiction of complex target interactions and uncovers central hub genes including *ESR1*, *TP53*, *AKT1*, *EGFR*, *TNF-α*, *HSP90AA*, and *HIF1A* (Fig. 1b).

### Pathway Enrichment Analysis of Common Targets for ATO, Resveratrol, and BC

To investigate the potential biological mechanisms of the 50 identified shared targets, we conducted analyses utilizing both the GO and KEGG pathway approaches. In Fig. 1c, among the top 20 enriched pathways analyzed, various pathways related to apoptosis exhibited notable upregulation. These pathways encompassed cellular response to chemical stimulus, regulation of programmed cell death, regulation of cell population proliferation, regulation of apoptotic process, apoptotic process, and response to oxygen-containing compound in GO. Furthermore, there was an increase in pathways such as pathways in cancer, ovarian steroidogenesis, steroid hormone biosynthesis, estrogen signaling pathway, endocrine resistance, and P13K-Akt signaling pathway in KEGG.

### ATO Curbs MCF-7 Cells Growth In Vitro

**Fig. 2:**
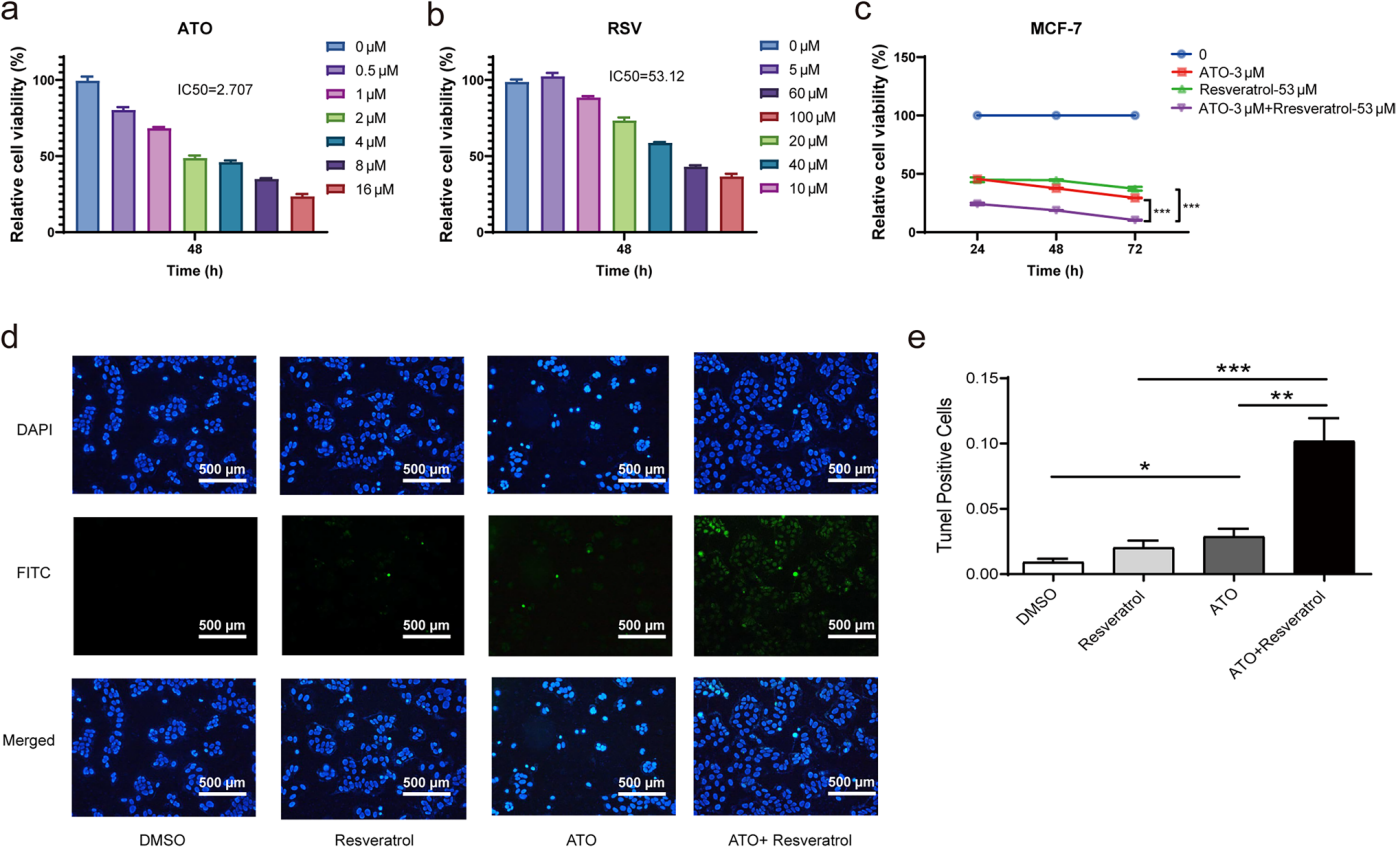
Observation of the impact of ATO and resveratrol on MCF-7 cell viability and apoptosis rates in MCF-7 cells with combined treatment. The image demonstrates the efficacy of treatments with varying concentrations of ATO and resveratrol on MCF-7 cells over a 48-h period. Treatment with either ATO or resveratrol alone resulted in a dose-dependent reduction in MCF-7 cell growth and proliferation (^***^p < 0.001 vs. 0 µM group) (**a**, **b**). Additionally, Fig. 2c illustrates that the combined use of ATO (3 µM) and resveratrol (53 µM) had a significantly greater impact on MCF-7 cell viability compared to using ATO or resveratrol individually (^***^p < 0.001). Apoptosis in MCF-7 cells was evaluated through the TUNEL assay after specified treatments. Representative images were captured for each experimental group, depicting TUNEL-positive cells in green stained with TUNEL (**d**), with quantification of TUNEL-positive cells provided (**e**). The data reported represents the mean ± SEM of three independent experiments conducted in triplicate, with statistical significance denoted as ^*^p < 0.05, ^**^p < 0.01, and ^***^p < 0.001 in comparisons between groups

We evaluated the survival rate of MCF-7 cells using the CCK-8 assay to explore the impact of ATO. MCF-7 cells were exposed to varying concentrations of ATO for a period of 48 h. A noticeable reduction in MCF-7 cell proliferation was observed as the concentration of ATO increased (Fig. 2a). After the 48-h exposure period, the half-maximal inhibitory concentration (IC50) of ATO for MCF-7 cell growth inhibition was calculated to be 2.707 µM.

### Resveratrol Curbs MCF-7 Cells Growth In Vitro

Our experiments on MCF-7 cells with resveratrol demonstrated a dose-dependent cessation of cell growth at the 48-h mark. At this point, the IC50 of resveratrol against MCF-7 cells was measured to be 53.12 µM (Fig. 2b).

### Dose of ATO and Resveratrol

The IC50 values for ATO and resveratrol were found to be 2.707 μM and 53.12 μM, respectively. These values denote the concentration needed to inhibit 50% of the activity of the target substance. This data is vital to understanding the potency of ATO and resveratrol in this study. A previous study (Zhao et al. 2014) found that 3 μM ATO and 50 μM resveratrol had a synergistic effect in MCF-7 cells over 48 h. Hence, a concentration of 3 μM ATO and 53 μM resveratrol for treatment of 48 h was selected for further studies.

### ATO-Resveratrol Combination Inhibits MCF-7 Proliferation

The growth of MCF-7 cells was analyzed by studying the effects of ATO (3 µM) and/or resveratrol (53 µM). We used the CCK-8 assay to check cell viability after 24, 48, or 72 h of treatment. Our observations showed a notable decrease in cell viability (*p* < 0.001) (Fig. 2c) when we combined both treatments, as opposed to using either ATO or resveratrol on their own. Using both ATO and resveratrol together has a significantly greater impact on the survival rate of MCF-7 cells compared to using either compound individually.

### ATO-Resveratrol Enhances Apoptosis in MCF-7 Cells

Our aim was to see how combined therapy affects MCF-7 cell apoptosis. We used the TUNEL assay to measure apoptosis. The apoptosis percentages for ATO plus resveratrol, ATO alone, resveratrol alone, and dimethyl sulfoxide (DMSO) were 10.203% ± 3.033%, 2.892% ± 1.031%, 2.021% ± 0.954%, and 0.928% ± 0.449%, respectively. Consequently, both the ATO and the combined-treatment groups demonstrated a significant rise in cell death compared to the DMSO group (*p* < 0.05 and *p* < 0.001, respectively). The combined treatment group exhibited a higher cell death ratio than both the ATO-only and resveratrol-only groups (*p* < 0.01) (Fig. 2d and e).

### ATO-Resveratrol Co-Regulates *ESR1*, *TP53*, and *AKT1* in MCF-7 Cells

**Fig. 3:**
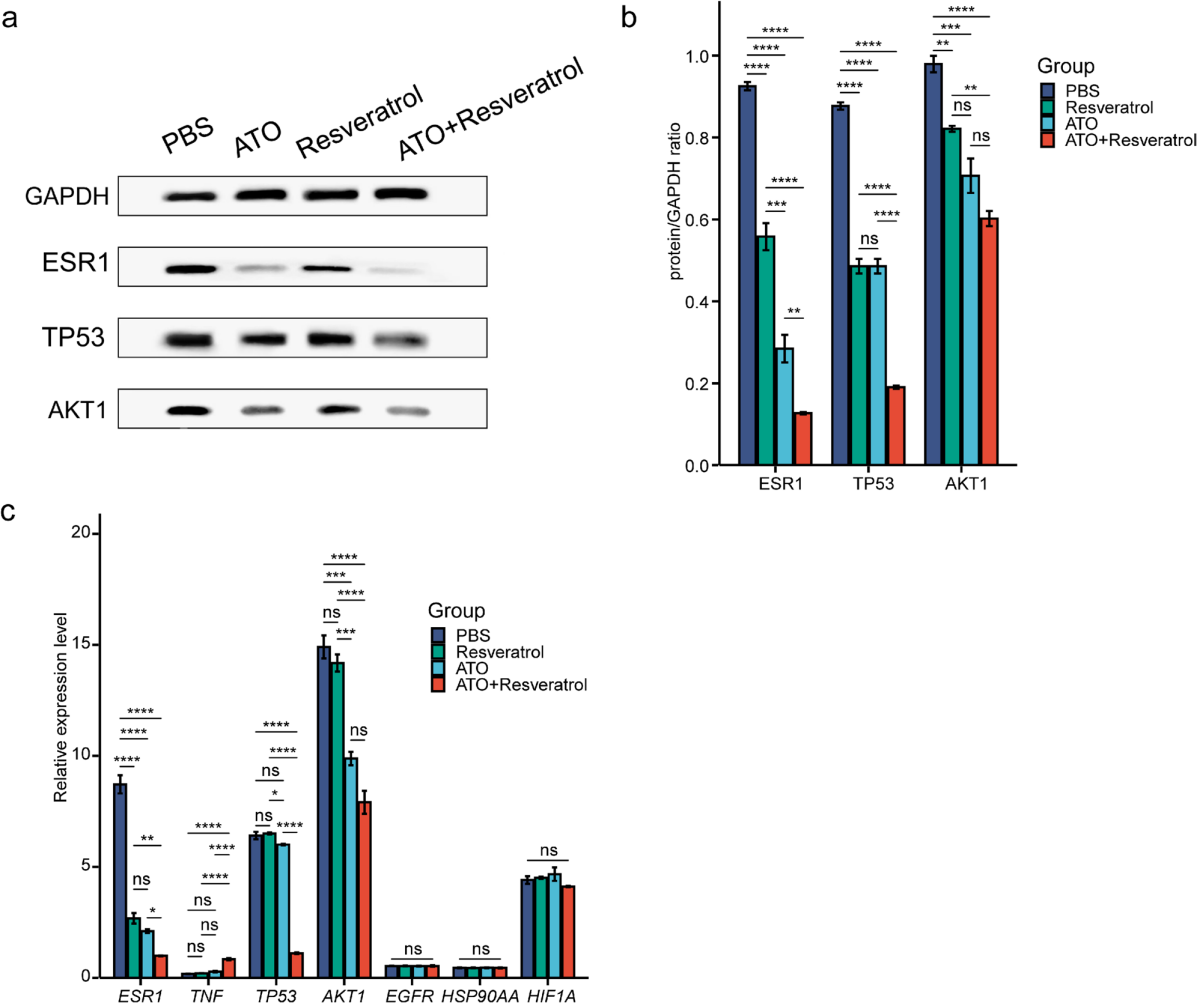
Evaluating the effect of combined ATO and Resveratrol therapy on *ESR1*, *TP53*, and *AKT1* gene expression in MCF-7 cells at mRNA and protein levels. The protein expression of ESR1, TP53, AKT1, EGFR, TNF, HSP90AA, and HIF1A was determined via Western blotting (**a**, **b**), while the mRNA expression levels of *ESR1*, *TP53*, *AKT1*, *EGFR*, *TNF*, *HSP90AA*, and *HIF1A* were evaluated using RT-qPCR (**c**). The presented data are the averages of three separate experiments, with error bars indicating the standard deviation. Statistical significance is indicated by ^*^p < 0.05, ^**^p < 0.01, ^***^p < 0.001, and ^****^p < 0.0001

The expressions of *ESR1*, *TP53*, *AKT1*, *EGFR*, *TNF-α*, *HSP90AA*, and *HIF1A* were assessed using RT-qPCR. The ATO + resveratrol group showed a more noticeable decrease in the expression of *ESR1*, *TP53*, *AKT1* compared to other groups (*p* < 0.05, *p* < 0.01, and *p* < 0.001, respectively) (Fig. 3c). Likewise, the combined therapy contributed to a significant decrease in the protein levels of ESR1, TP53, and AKT1 in MCF-7 cells (Fig. 3a and b).

## Discussion

ATO, a traditional Chinese medicine, is commonly used as an anti-leukemia agent in China. Evidence suggests ATO may also treat liver, breast, and ovarian cancer (Chen et al. 2023a; Nasrollahzadeh et al. 2020; Tang et al. 2022; Zhang et al. 2021). However, its potential to induce cardiomyopathy limits its clinical application (Li et al. 2022a). The risk of cardiac toxicity is dose-dependent (Li et al. 2022a; Vineetha and Raghu 2019). Discovering a component that protects the heart while enhancing ATO's anticancer effects could be crucial. Resveratrol may reduce cardiac toxicity (Akgun-Unal et al. 2023; Xia et al. 2017) and alleviate heart-related side effects of ATO (Chen et al. 2015; Fan et al. 2014; Mondal et al. 2018). Further exploration indicates that resveratrol could potentially enhance the therapeutic impact of ATO on breast cancer (Zhao et al. 2014). However, the exact mechanism of how resveratrol enhances the effect of low-concentration ATO is currently not well understood.

In our study, we explored the synergistic effects of ATO and resveratrol on BC through network pharmacology and experimental validation. Our analysis identified 50 shared targets among ATO, resveratrol, and BC, suggesting that their combined application could impact multiple targets crucial to BC. GO and KEGG pathway analyses highlighted significant upregulation of apoptosis pathways among the top 20 enriched pathways. Key genes such as *ESR1*, *TP53*, and *AKT1*, alongside *EGFR*, *TNF-α*, *HSP90AA*, and *HIF1A*, emerged as central to the therapeutic response to ATO and resveratrol in BC. Subsequent vitro experiments confirmed these findings, demonstrating that the combination of ATO and resveratrol effectively regulated the expression of *ESR1*, *TP53*, and *AKT1* in MCF-7 cells, surpassing the effects of individual treatments.

The *ESR1* gene codes for the alpha estrogen receptor, a crucial protein involved in the growth and advancement of BC (Li et al. 2022b; Veiga et al. 2022). Previous research suggests that increased expression of in breast cancer increases the likelihood of developing estrogen receptor (ER) positive breast cancer in women after menopause (Furth et al. 2023b; Li et al. 2022b). Nevertheless, the risk may be mitigated by anti-hormone drugs such as tamoxifen and letrozole (Furth et al. 2023a). The joint intervention of ATO and resveratrol displays encouraging outcomes in reducing the expression of the *ESR1* gene and ESR1 protein, suggesting its potential as a successful therapy for ER positive BC.

*TP53*, also referred to as tumor protein p53, has a vital function in regulating the cell cycle (Soussi and Wiman 2015). It primarily triggers apoptosis, or programmed cell death (Soussi and Wiman 2015). When the *TP53* gene mutates, these regulation systems malfunction, causing rampant cell growth—a typical characteristic of various cancers (Mandinova and Lee 2011). *TP53* mutations are present in 80% of cases of triple-negative breast cancer (TNBC), which refers to tumors that do not express ER, PR, and ErbB2/HER2 (Liu et al. 2018a). TNBC is notably challenging to treat as it doesn't react to endocrine or HER2 monoclonal drug treatment (Green-Tripp et al. 2022). Nevertheless, the concurrent administration of ATO and resveratrol leads to a notable reduction in both the *TP53* gene and TP53 protein expression. This suggests that this combination could potentially be a promising treatment for *TP53* mutant breast cancer.

*AKT1*, identified as a proto-oncogene, tends to be overexpressed or unusually activated in an array of human cancers. Notably, about 40% of BC show enhanced *AKT1* activity (Liu et al. 2018b). This is primarily due to mutations and amplification of the *AKT1* gene, with *E71K* mutation being the most prevalent. The mutation modifies two crucial elements of *AKT1*, strengthening its connection with PI (3,4,5) P3 lipids and facilitating its ubiquitination. Both modifications induce atypical elevation in *AKT1* membrane positioning and phosphorylation at Thr308, resulting in atypical carcinogenic capacity (Ward et al. 2011). The strategy of hindering the PI3K/mTOR pathway by targeting AKT1 has long been a focus for innovative anti-tumor drug development. Nonetheless, the intricate pathway feedback signals involved in AKT1 make it a challenge to develop drugs that are both highly effective and low in toxicity. To date, no AKT1 inhibitor has gained global marketing approval. The first batch of AKT-targeted drugs for breast cancer treatment includes Capivasertib (Howell et al. 2022; Turner et al. 2019), a highly selective inhibitor that targets three AKT subtypes (AKT1/2/3) and disrupts the signals that cancer cells use to divide and grow. ATO + resveratrol combined treatment can significantly reduce *AKT1* gene and AKT1 protein expression, indicating that this combination treatment could potentially serve as a potent AKT1 inhibitor for breast cancer in the future.

Inhibitors targeting ESR1, TP53, and AKT1 represent significant advancements in the treatment of BC. However, achieving effective disease control remains challenging for some patients due to factors such as treatment ineffectiveness, non-adherence, adverse effects, or intolerance. This underscores the need for alternative therapeutic strategies. ATO and resveratrol have shown the ability to inhibit key genes, including *ESR1*, *TP53*, and *AKT1*, which are crucial in BC progression. Additionally, resveratrol's cardioprotective properties may help mitigate the cardiovascular damage often associated with ATO treatment (Fan et al. 2014). By simultaneously targeting multiple pathways involved in apoptosis, the combination of ATO and resveratrol may potentially overcome resistance mechanisms that limit the effectiveness of single-target inhibitors. Nonetheless, further research and clinical trials are essential to validate these findings and fully assess the therapeutic potential of ATO and resveratrol in managing BC.

Beyond BC, the combined use of ATO and resveratrol has shown promising therapeutic benefits in lung adenocarcinoma and leukemia (Chen et al. 2019; Gu et al. 2016). Studies have demonstrated that this combination can effectively inhibit cell proliferation and induce apoptosis through various mechanisms. One key mechanism involves the downregulation of several anti-apoptotic genes, including *Bcl-2*, *NF-κB*, and *TP53*, which is consistent with some of our research findings (Chen et al. 2019). Additionally, the accumulation of reactive oxygen species (ROS) triggers apoptosis via a mitochondria-dependent pathway (Gu et al. 2016). This pathway is crucial for initiating the apoptotic process by disrupting mitochondrial membrane potential and releasing pro-apoptotic factors. Given these findings, there is significant interest in further exploring the combined use of ATO and resveratrol in BC. Future research should focus on elucidating the precise molecular mechanisms involved in their synergistic effects and determining optimal dosing regimens to maximize therapeutic outcomes.

## Conclusions

Our research results show that the key targets of combined treatment with ATO and resveratrol include *ESR1*, *TP53*, and *AKT1*. However, whether the expression of these genes is correlated with their associated biological functions requires further investigation. Moving forward, it is essential to conduct more in vivo studies to validate these promising findings.

## Data Availability

We confirm that the data which supports the findings of this study is available within the article and/or its supplementary materials.

## Abbreviations

ATO: Arsenic trioxide
BC: Breast cancer
ESR1: Estrogen receptor 1
TP53: Tumor protein P53
AKT1: V-AKT murine thymoma viral oncogene homolog 1
APL: Acute promyelocytic leukemia
PVDF: Polyvinylidene difluoride
EGFR: Epidermal growth factor receptor
TNF-α: Tumor necrosis factor-alpha
HSP90AA: Heat shock protein 90 kDa alpha
HIF1A: Hypoxia-inducible factor 1A
IC50: Half‐maximal inhibitory concentration
